# NKG2H-Expressing T Cells Negatively Regulate Immune Responses

**DOI:** 10.3389/fimmu.2018.00390

**Published:** 2018-03-01

**Authors:** Daniela Dukovska, Daniel Fernández-Soto, Mar Valés-Gómez, Hugh T. Reyburn

**Affiliations:** ^1^Department of Immunology and Oncology, National Centre for Biotechnology, CSIC, Madrid, Spain

**Keywords:** NKG2H, T cells, negative regulation, DAP12, cell–cell interaction

## Abstract

The biology and function of NKG2H receptor, unlike the better characterized members of the NKG2 family NKG2A, NKG2C, and NKG2D, remains largely unclear. Here, we show that NKG2H is able to associate with the signaling adapter molecules DAP12 and DAP10 suggesting that this receptor can signal for cell activation. Using a recently described NKG2H-specific monoclonal antibody (mAb), we have characterized the expression and function of lymphocytes that express this receptor. NKG2H is expressed at the cell surface of a small percentage of peripheral blood mononuclear cell (PBMC) and is found more frequently on T cells, rather than NK cells. Moreover, although NKG2H is likely to trigger activation, co-cross-linking of this receptor with an NKG2H-specific mAb led to decreased T cell activation and proliferation in polyclonal PBMC cultures stimulated by anti-CD3 mAbs. This negative regulatory activity was seen only after cross-linking with NKG2H, but not NKG2A- or NKG2C-specific monoclonal antibodies. The mechanism underlying this negative effect is as yet unclear, but did not depend on the release of soluble factors or recognition of MHC class I molecules. These observations raise the intriguing possibility that NKG2H may be a novel marker for T cells able to negatively regulate T cell responses.

## Introduction

The NKG2 family of receptors are type II transmembrane glycoproteins containing an extracellular C-type lectin-like domain, encoded in a gene cluster on human chromosome 12p12-p13 and mainly expressed by NK cells and subsets of T cells (1). NKG2 family receptors are divided into inhibitory and activating forms (2, 3). NKG2A/2B receptors contain cytoplasmic immunoreceptor tyrosine-based inhibitory motifs (ITIM) and function as inhibitory receptors (4). By contrast, NKG2C, NKG2F, and NKG2E/2H receptors are generally classed as activating receptors, since they contain a lysine residue in the transmembrane region allowing association with the ITAM-containing adapter protein DAP12 (5). NKG2D protein also has a charged amino acid in the transmembrane domain, but in humans only associates with the signaling adapter molecule DAP10 and not DAP12 (6, 7). The biology and functions of the NKG2A, NKG2C, and NKG2D receptors in immune responses to infections and in cancer have been studied extensively (8, 9); however, only very little is known about the biology of the other members of this family: NKG2F, NKG2E, and NKG2H.

The NKG2F gene encodes a receptor with a truncated extracellular domain without a lectin-like domain (10) and, although NKG2F protein is expressed in NK cells and can associate with DAP12, this receptor is only found intracellularly (11). It is not clear if this receptor has any function in NK cell activation or whether it is simply a “fossil” within the NKG2 gene family.

NKG2E mRNA has been detected in NK cells and CTL (12–15), but, due to the lack of a specific antibody, expression of NKG2E protein has not been demonstrated. Soluble recombinant NKG2E protein, in complex with CD94, has been reported to bind HLA-E (16); however in transfected cells, NKG2E did not reach the cell surface, despite associating with CD94 and DAP12 (17).

NKG2H is an alternative splice form of NKG2E in which intron VI is not removed and exon VII is not present. This alteration deletes the sequence responsible for the intracellular retention of NKG2E and gives rise to a specific 33 amino acid sequence located at the membrane distal portion of the extracellular domain of the NKG2H protein. Previous studies have demonstrated that generation of the splice variants NKG2E and -H seems to occur at a constant ratio (12) and that mRNA for NKG2H can be found in both NK cells and CTL (12, 18). Like NKG2C and NKG2E, NKG2H has a short cytoplasmic tail and lacks ITIMs, but does possess a charged residue in the transmembrane domain that could permit interaction with the DAP12 adapter molecule. The only previous description of NKG2H expression analyzed a TCRαβ^+^ CD8^+^ T cell clone and reported that NKG2H was an activating receptor able to trigger redirected lysis in a TCR-independent manner (18). However, further progress in understanding the biology and function of the orphan NKG2H receptor including signaling, molecular interactions, and ligand binding has been seriously hampered due to lack of monoclonal antibodies specific for this molecule.

We now show that NKG2H associates with both the DAP10 and DAP12 signaling adapter molecules, suggesting that NKG2H will act as an activating receptor. Using a new commercially available monoclonal antibody (mAb) specific for NKG2H, we have carried out the first study of the phenotype and functional capacity of lymphocytes expressing the NKG2H receptor. These data show that the NKG2H receptor is expressed on low numbers of peripheral blood lymphocytes, but that activation of these NKG2H-expressing cells markedly reduces the activation and proliferation of other T cells in the culture. Although the mechanism of action of this receptor is as yet unknown, we have also shown that this suppressive activity did not depend on the release of soluble factors or recognition of MHC class I molecules.

## Materials and Methods

### Transfection of 293T Cells with NKG2H Constructs

A construct encoding full length NKG2H tagged with a c-myc epitope at the N-terminus was prepared by PCR using the oligonucleotides 5′-cgggatccgccaccatggcatcaatgcagaagctgatctcagaggaggacctgaataaacaaagaggaaccttctc-3′ and 5′-ttttccttttgcggccgcttatgcaatcataatatcatttctg-3′ and cloned into the pHR-sin vector (19). Plasmids encoding CD94, FLAG-DAP12 (gifts of C. Chang), and DAP10-GFP have been described previously (20–22). 293T cells were transfected with the indicated plasmids using the JETPEI reagent according to the manufacturer’s recommendations.

### Reagents

The 9E10 mAb (ATCC CRL-1729), specific for the human c-myc gene product, was purified from hybridoma supernatant. FLAG-tag and GFP-specific antibodies were purchased from Sigma-Aldrich and Santa Cruz Biotechnology.

Conjugated antibodies for blood lymphocyte subpopulations were from BioLegend and Beckman Coulter, as follows: CD3-FITC (Clone UCHT1); CD3-PacificBlue (Clone OKT3); CD4-APC (Clone OKT4); CD8-PeCy7 (Clone RPA-T8); CD45RA-ECD (Clone 2H4LDH11LDB9); CD45RO-FITC (Clone UCHL1); CD56-PE (Clone MEM-188); CD69-APC/Cy7 (Clone FN50); CD56-PerCP/Cy5.5 (Clone N901). Monoclonal antibodies specific for NKG2H (Clone 633810), NKG2A (Clone 131411), and NKG2C (Clone 134591) were purchased from R&D Systems (Abingdon, UK). To validate the specificity of the commercial NKG2H mAB, we stained the NKL cell line transfected, or not, with an NKG2H cDNA cloned in the into the lentiviral vector pHRSIN-C56W-UbEM (a gift of Prof Paul Lehner, Cambridge Institute for Medical Research, Cambridge) to simultaneously express NKG2H under the SFFV promoter and the GFP derivative protein, Emerald, under a ubiquitin promoter. Untransfected NKL cells stain positively for NKG2A, NKG2C, and NKG2D expression, but only bind the NKG2H mAb after lentiviral transduction with the NKG2H cDNA (Figure S1 in Supplementary Material).

Purified human anti-CD3 (ATCC, Clone CRL-8001) and anti-CD28 (BD Biosciences, Clone CD28.2) antibodies were used in the immobilized stimulation assays. Secondary PE-conjugated anti-mouse Ig antibody was purchased from DakoCytomation. Blocking MHC class I-specific antibody (HP-1F7), a gift of Dr. Miguel López-Botet (UPF, Barcelona), has been described previously (23). Annexin-V-FITC and 7AAD were purchased from Beckman Coulter. The isotype controls used in the flow cytometry and functional experiments were mouse MOPC-21 (IgG1, purchased from Sigma) and OX-68 (IgG2a, ECACC Hybridoma Collection: OX-68).

### Western Blot and Immunoprecipitation

Cells were lysed in 1% digitonin (in 0.12% Triton X-100, 150 mM NaCl, 20 mM Triethanolamine, pH 7.8, and 0.02% sodium azide). After centrifugation to remove insoluble material and preclearing, the indicated antibodies were added to the lysates to a final concentration of 5 µg/ml. After incubation on ice, the immune complexes were recovered using Protein G sepharose and analyzed by SDS-PAGE and western blotting using specific antibodies.

### Peripheral Blood Mononuclear Cell (PBMC) Culture

This study was carried out in accordance with the recommendations of the ethical committee of the CSIC, Madrid, with written informed consent from all subjects. All subjects gave written informed consent in accordance with the Declaration of Helsinki. The protocol was approved by the local ethical committee of the Regional Transfusion Centre, Madrid.

Peripheral blood mononuclear cells were isolated from anonymous healthy volunteers by Ficoll-Hypaque (GE Healthcare) density gradient centrifugation. Purified PBMCs were cultured in RPMI supplemented with 10% human serum (HS) (Sigma Aldrich), 2 mM l-glutamine, 10 mM HEPES, 100 U/ml penicillin, 100 U/ml streptomycin, and 50 µM β-mercaptoethanol and incubated in a humidified incubator at 37°C and 5% CO_2_.

### Flow Cytometry

For immunofluorescence staining of PBMCs, cells were washed with PBS containing 1% FBS, 0.5% BSA, and 0.05% sodium azide (PBA), pre-treated with 1% normal HS to block non-specific binding of mAbs to FcR. Subsequently, PBMC were stained with the unlabeled NKG2H-specific mAb, or IgG2a isotype control, and PE-labeled F(ab′)2 fragments of goat anti-mouse Ig (Dako). These samples were then blocked with 10% normal mouse serum before being stained with directly labeled mAbs specific for other markers (CD3, CD4, CD8, etc.). Samples were analyzed using Gallios (Beckman Coulter) or Cytomics FC500 (Beckman Coulter). Flow cytometry data were analyzed using either Kaluza v1.2 or FlowJo V9.6.2 software.

### Stimulation of PBMC Using Immobilized Antibody

To immobilize the antibodies used for stimulation, 24-well plates were coated with purified human anti-CD3, anti-CD3/CD28, or anti-CD3/NKG2H mAbs in PBS and incubated for 2–3 h at RT. The mAb concentration is specified in each experiment. Unbound mAb was removed by washing with PBS. 1.5–2.10^6^ PBMCs per well were added to the mAb coated plates in RPMI containing 10% HS and incubated for 48 or 72 h at 37°C and 5% CO_2_ in a humidified incubator. After this period of incubation, cells were collected and analyzed by flow cytometry.

### Proliferation Assay

Peripheral blood mononuclear cells were resuspended in RPMI and incubated for 5–7 min at 37°C in the incubator with the intracellular fluorescent dye CFSE at a final concentration of 2.5 µM. After two washes, CFSE-labeled PBMCs were cultured in 24-well plates to which mAb had been pre-absorbed as described. Flow cytometry analyses of CFSE-labeled cells were performed on days 2, 3, and 4 after stimulation.

### Cell Viability Assay

Peripheral blood mononuclear cells stimulated using immobilized antibodies were pre-stained, washed with PBS, and stained with Annexin-V FITC and 7AAD in annexin-binding buffer (Invitrogen) following the manufacturer’s instructions.

### Supernatant Transfer Experiments

For supernatant transfer experiments, PBMCs from healthy donors were stimulated with the indicated immobilized antibodies for 48 h, after that, the supernatant was collected and frozen at −20^o^C (“donor culture”). PBMCs from healthy donors were purified and stimulated on plates pre-coated with CD3-specific mAb (“recipient culture”). Stored supernatant from the “donor cultures” was diluted 1:2 or 1:5 in complete RPMI medium supplemented with 10% HS and added to the corresponding “recipient culture.” PBMCs stimulated with immobilized anti-CD3 antibody in complete RPMI supplemented with 10% HS were used as a control. After 48 h, the percentage of CD69-expressing cells was determined by flow cytometry.

## Results

### NKG2H Can Pair with both the DAP10 and DAP12 Signaling Adapter Molecules

**Figure 1:**
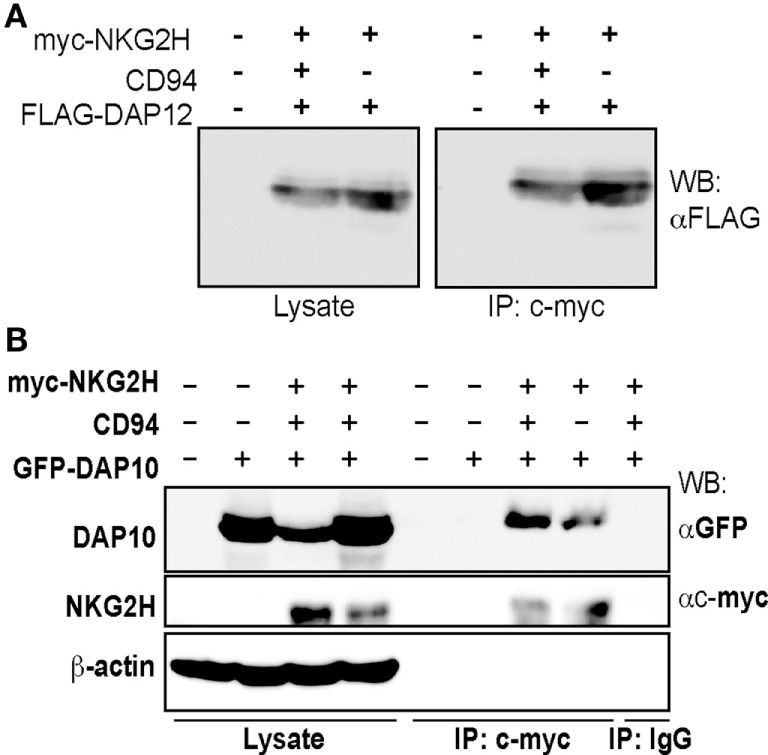
NKG2H can associate with both the DAP12 and DAP10 signaling adapter molecules. 293T cells were transfected with expression vector encoding N-terminally c-myc tagged NKG2H, wild-type CD94, and DAP12 **(A)** or DAP10 **(B)** as indicated and cell lysates were prepared. Samples were immunoprecipitated with either an isotype control or the c-myc-specific 9E10 mAb and then 20 µg of the whole cell lysates or the entire immunoprecipitates were analyzed by western blot using the indicated antibodies. The data are representative of three to five experiments.

The high degree of sequence similarity between NKG2C, NKG2E, and NKG2H (Figure S2 in Supplementary Material) led to the prediction that NKG2H would associate with the adapter molecule DAP12 to form an activating immunoreceptor complex (18). To investigate this possibility, we transfected 293T cells with a c-myc tagged NKG2H construct in the presence and absence of CD94 and FLAG-tagged DAP12. Western blot analysis of anti-myc immunoprecipitates of transfected cells, lysed in 1% digitonin buffer, confirmed that NKG2H associates with DAP12 and showed that this did not depend on CD94 (Figure 1A). Surprisingly, NKG2H was also observed to associate with the DAP10 adapter molecule (Figure 1B). These data strongly suggested that NKG2H was likely to be an activating receptor; therefore, the next set of experiments addressed the issues of which immune cells expressed this receptor and what were the functional consequences of receptor ligation.

### NKG2H Is Mainly Expressed by Subpopulations of T Cells in Healthy Donors

**Figure 2:**
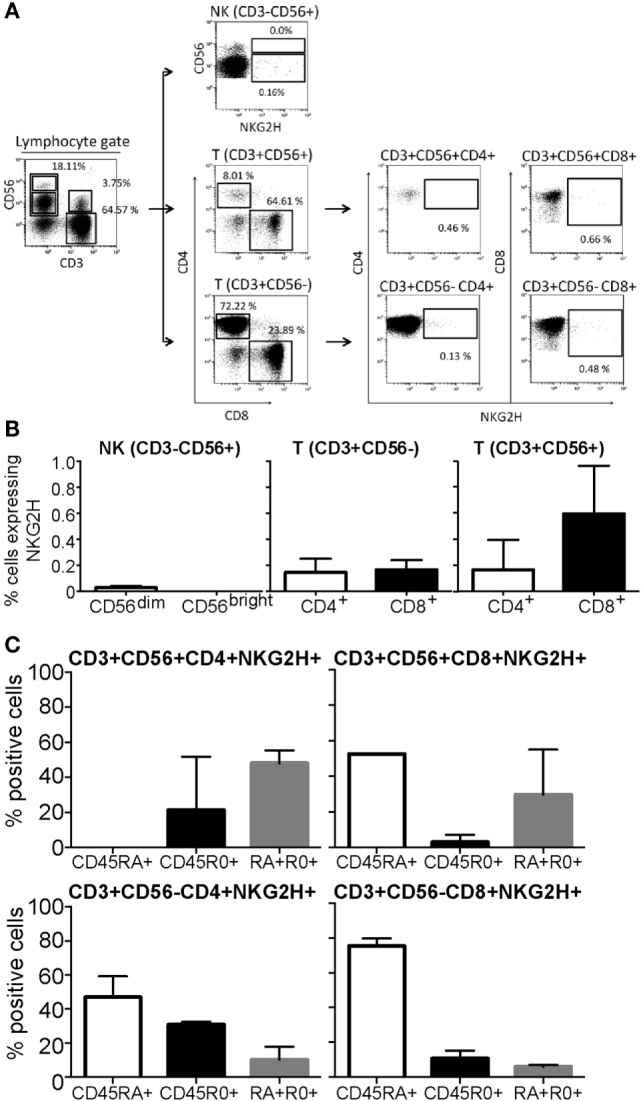
Expression of NKG2H in peripheral blood mononuclear cells (PBMCs) from healthy donors. PBMCs isolated from healthy individuals were analyzed by flow cytometry. **(A)** Gating strategy. NK cells were defined by gating the CD3^−^CD56^+^ cells within the lymphocyte region, defined by forward and side scatter parameters. CD56^high^ and CD56^dim^ NK subsets were analyzed separately. For T cells, CD8^+^ and CD4^+^ T cells were analyzed within the CD3^+^CD56^−^ region. CD3^+^CD56^+^ double positive effector lymphocytes were also gated as CD3^+^CD56^+^CD8^+^ and CD3^+^CD56^+^CD4^+^. The percentage of NKG2H-positive cells was analyzed within each region. **(B)** The percentage of NKG2H^+^ cells in the different lymphocyte populations was determined in PBMCs from five different healthy individuals. Data show the mean and SD of independent experiments after subtraction of isotype control values. **(C)** The percentage of NKG2H^+^ cells expressing CD45RA, CD45RO, or both CD45RA and RO in different populations of T cells was determined in PBMCs from five different healthy individuals. Data show the mean and SD of independent experiments.

Flow cytometry analysis of freshly isolated PBMCs from a panel of healthy individuals was used to characterize the cell populations expressing NKG2H at the cell surface. A five-color staining strategy, using a combination of anti-CD3, CD4, CD8, CD56, and anti-NKG2H mAbs was used to analyze NKG2H expression on the different PBMC subsets defined by these surface markers. The gating strategy for these analyses is shown in Figure 2A.

Despite some variation between blood donors, NKG2H was consistently found expressed on the surface of low numbers of PBMCs (Figure 2B). Little or no NKG2H receptor was expressed by freshly isolated NK cells, either the CD56^dim^ or CD56^bright^ subsets. However, both CD3^+^CD56^+^ and CD3^+^CD56^−^ T cells expressed NKG2H, particularly CD8^+^CD56^+^T cells. It is interesting to note that NKG2H was first identified and cloned from a human CD8^+^ T cell clone (18). These data were confirmed in independent experiments using polyclonal sera obtained by immunization of mice with a GST-fusion protein expressing 33 C-terminal amino acids of NKG2H (Figure S3 in Supplementary Material). As expected, preincubation of the NKG2H-specific mAb with peptide antigen markedly reduced the staining observed (Figure S4 in Supplementary Material).

Expression of CD45RA and CD45RO splice isoforms is often used to analyze the differentiation state of T cells, CD45RA denoting naïve/effector T cells and CD45RO denoting memory T cells (24). Analysis of CD45RA and RO expression by NKG2H^+^ cell subsets revealed that, although the CD45RO antigen was present on some NKG2H^+^ T cells, the vast majority of these cells expressed the CD45RA marker (Figure 2C). Consistent with published data (25), NK cells, whether NKG2H^+^ or not, expressed the CD45RA isoform almost exclusively (not shown).

### Activation of NKG2H Expressing Cells Markedly Reduces the Activation of Other T Cells in the Culture

**Figure 3:**
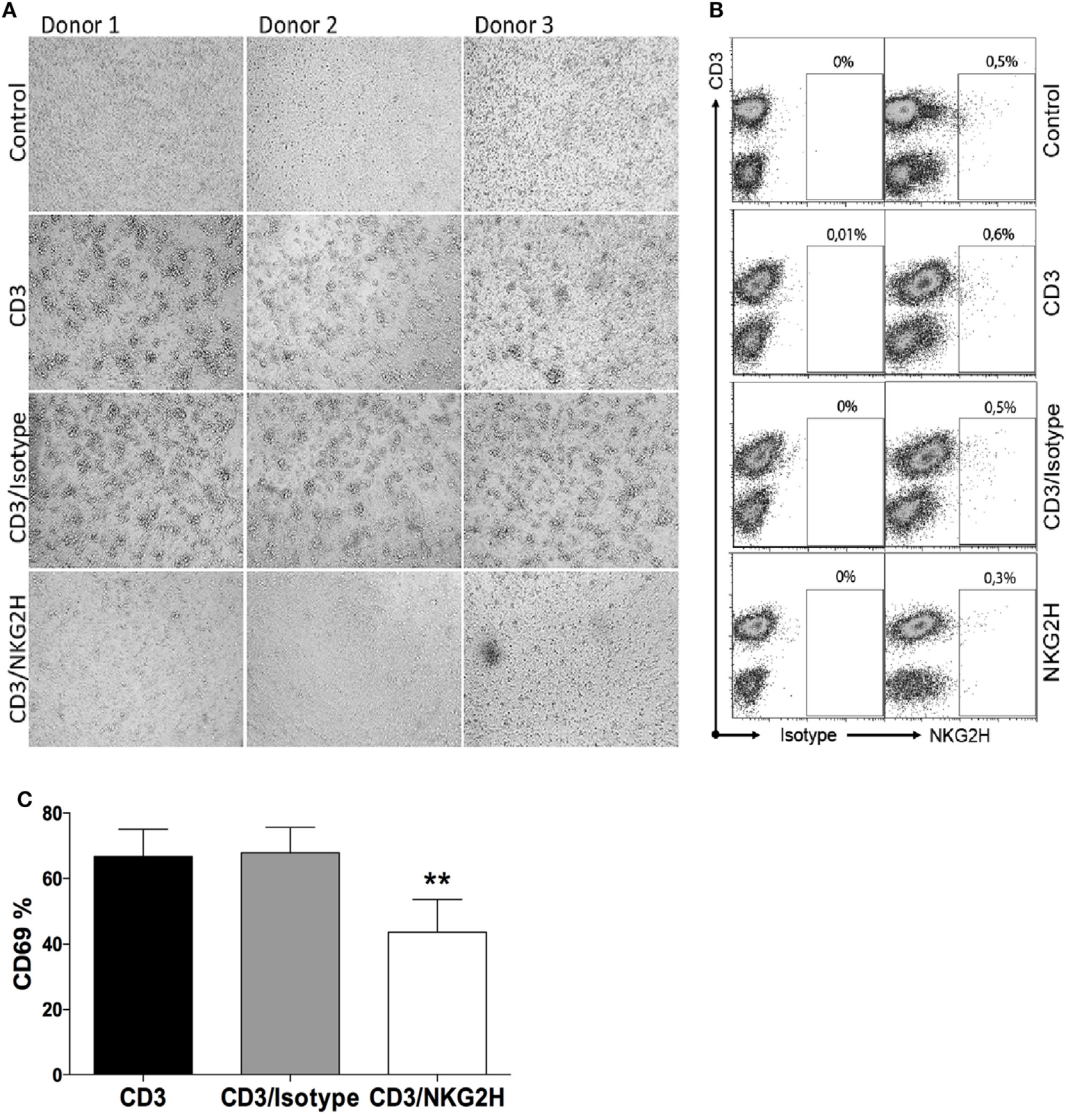
Lymphocyte activation is reduced after cross-linking peripheral blood mononuclear cells (PBMCs) with antibodies directed against CD3 and NKG2H. **(A)** Microscope images of PBMCs from three representative healthy donors incubated in plates pre-coated with anti-CD3 (1 µg/ml), anti-CD3/IgG2a control, or anti-CD3/NKG2H (1/10 µg/ml) antibodies for 48 h. No coating (PBS) was used as a negative control. **(B)** NKG2H expression on T cells in the cultures under different stimulation conditions. **(C)** Quantitative analysis of the percentage of activated cells (expressing CD69) after anti-CD3/IgG2a control or anti-CD3/NKG2H stimulation. Mean data of five independent experiments are represented; *p < 0.05; **p < 0.01.

The association of NKG2H with the signaling adapter molecules DAP12 and DAP10 suggests that NKG2H will likely be an activating receptor. This idea is consistent with previous observations that engagement of NKG2H could trigger both early activation events such as Ca^2+^ mobilization, and late effector functions such as induction of TCR-independent cytolytic activity and IFN-γ production in a T cell clone maintained in IL-2 (18). To establish the functional consequences of ligation of the NKG2H receptor on freshly isolated resting T cells, PBMCs were exposed to a suboptimal dosage of immobilized anti-CD3 antibody in combination with anti-NKG2H antibody for 48 h. As controls, PBMCs were incubated either with immobilized anti-CD3 antibody alone or a combination of anti-CD3 with an isotype control IgG2a antibody. Strikingly, simple inspection by light microscopy revealed that those cell cultures stimulated with the combination of CD3 and NKG2H-specific mAbs appeared far less activated than the control cells after 48 h of stimulation (Figure 3A), and these differences were much more pronounced after 72 h (not shown). Flow cytometry analysis confirmed that only a small proportion of T cells in these cultures expressed NKG2H at the cell surface (Figure 3B); nevertheless, consistent with the observations made by light microscopy, there was a significant decrease of the percentage of CD69-expressing cells in cultures stimulated with anti-CD3/NKG2H antibodies compared to cultures stimulated only with anti-CD3 (Figure 3C). In control experiments, co-incubation of the anti-CD3 mAb with other mAbs, including the NKG2H-specific mAb had no significant effect on the amount of anti-CD3 mAb immobilized (Figure S5 in Supplementary Material).

Since only a small proportion of PBMCs express NKG2H at the cell surface, these results suggest that activation of NKG2H-expressing T cells might negatively regulate the activation and/or proliferation of other T cells in the culture.

### Activation of NKG2H Expressing Cells Markedly Reduces the Proliferation of Other Cells in the Culture

**Figure 4:**
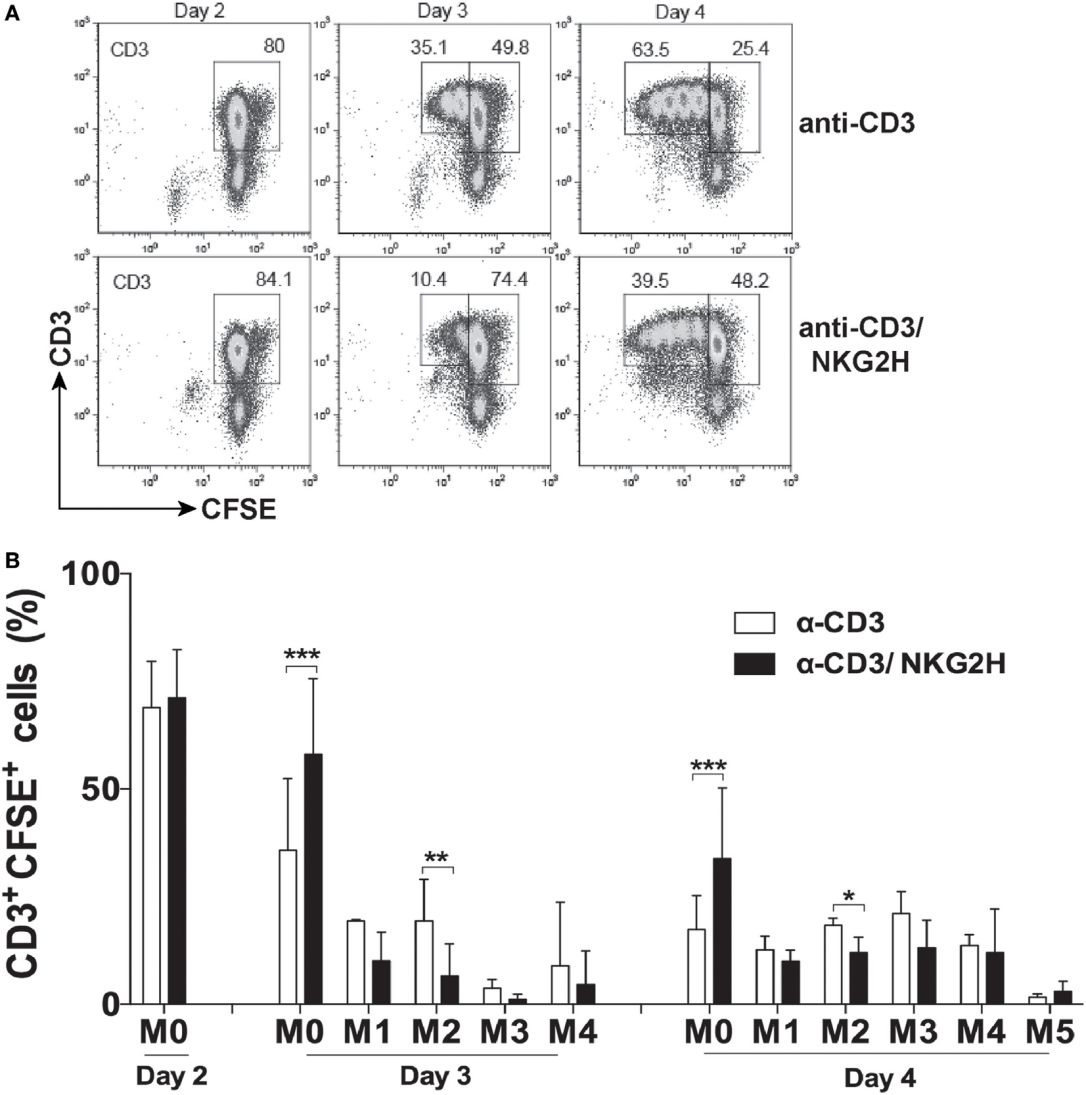
NKG2H stimulation reduces proliferation of lymphocytes in the cell culture. **(A)** Representative flow cytometry histograms of CFSE-labeled peripheral blood mononuclear cells (PBMCs) stimulated with anti-CD3 (1 µg/ml) or anti-CD3 and NKG2H (1/10 µg/ml, respectively) antibodies for 2, 3, or 4 days. Leukocytes were gated on forward and side scatter, and the plots show CD3 staining vs. CFSE fluorescence. **(B)** Representation of the proliferation index. Mean data and SDs of several independent experiments with six different donors are represented; *p < 0.05; **p < 0.01, ***p < 0.001. M0: no mitosis, M1–M5: number of mitotic divisions.

To elucidate whether the decrease of CD69 expression on T cells, after NKG2H stimulation, was accompanied by growth changes in the PBMC culture, T cell proliferation was also analyzed. CFSE-labeled PBMCs were exposed to immobilized anti-CD3/NKG2H or control anti-CD3 antibodies and cell proliferation was evaluated on days 2, 3, and 4 of the culture. As shown in Figures 4A,B, the proliferation of T cells in cultures stimulated with mAbs to CD3 and NKG2H was significantly reduced compared to control cells. Thus, co-cross-linking of TCR/CD3 and NKG2H on the small fraction of T cells expressing the latter receptor, significantly inhibited the activation and proliferation of the majority of T cells in the culture.

### Ligation of NKG2H, but Not NKG2A or NKG2C, Impairs PBMC Activation

**Figure 5:**
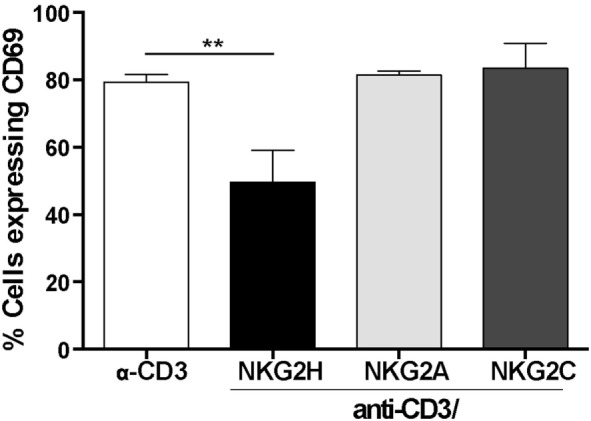
NKG2H, but not NKG2A or NKG2C cross-linking leads to decreased lymphocyte activation. 24-well plates were coated with anti-CD3 (1 µg/ml), anti-CD3/NKG2A (1/10 µg/ml), anti-CD3/NKG2C (1/10 µg/ml), or anti-CD3/NKG2H (1/10 µg/ml) antibodies. Purified peripheral blood mononuclear cells (PBMCs) from healthy donors were isolated and cultured in antibody-coated plates for 48 h. After this time, the cells were collected, stained with anti-CD69 antibody and analyzed by flow cytometry. Analyses are gated on leukocytes (based on forward and side scatter). Representative data of three experiments from two different donors are shown; **p < 0.01.

Both, the inhibitory CD94/NKG2A and activating CD94/NKG2C receptors have been described to be expressed and functional in αβ and γδ CD8^+^ T cells (13, 15, 26). Accordingly, to test whether the observed reduction in T cell activation after anti-CD3/NKG2H cross-linking was a specific feature of NKG2H receptor signaling or could also be produced after stimulation by other NKG2 family members, the previous experiments were replicated using PBMCs cultured in plates where combinations of anti-CD3/NKG2C and anti-CD3/NKG2A antibodies had been immobilized. As previously, simultaneous exposure to anti-CD3 and anti-NKG2H antibody significantly decreased the percentage of T cells expressing the activation marker CD69. In contrast, treatment with anti-CD3/NKG2C or anti-CD3/NKG2A had no significant effect on T cell activation compared to PBMCs stimulated only with anti-CD3 (Figure 5). Altogether, these observations support that stimulation through NKG2H, but not NKG2A or NKG2C, specifically leads to reduced T cell activation in the culture. These experiments also argue against the idea that the reduced CD3-induced activation seen after co-ligation with NKG2H is due to a reduction in the efficiency of immobilization of anti-CD3 mAb in the presence of a 10-fold excess of another mAb.

### Activation of NKG2H^+^, but Not NKG2A^+^ or NKG2C^+^, Cells Is Associated with the Induction of Cell Death in the Culture

**Figure 6:**
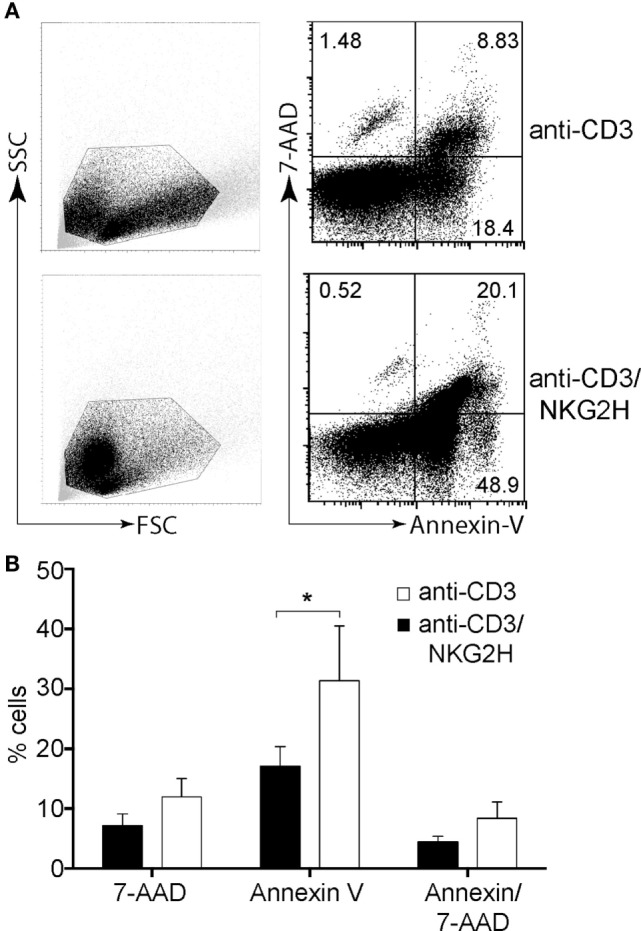
NKG2H stimulation results in decreased cell survival. Purified peripheral blood mononuclear cells (PBMCs) were activated on plates coated with anti-CD3 (1 µg/ml) or anti-CD3/NKG2H (1/10 µg/ml) antibodies for 48 h. In parallel, stimulation of PBMCs using immobilized anti-CD3 (1 µg/ml), and the combination of anti-CD3/NKG2A and anti-CD3/NKG2C antibodies (1/10 µg/ml) was also carried out (data not shown). Cell survival was analyzed by Annexin V/7AAD staining. **(A)** Representative flow cytometry dot plots **(B)** Changes in the percentages of cells positive for Annexin V and 7AAD were calculated. Mean data and SDs of six experiments with different healthy donors are represented; *p < 0.05.

The previous observations raised the question as to how activation of the 0.5–1% of T cells expressing NKG2H could modulate the activation of the remaining lymphocytes that had no appreciable expression of NKG2H. One possible solution to this conundrum was that activation of the minority NKG2H-expressing population could result in the induction of death of other T cells in culture. To explore whether the reduced proliferation of PBMCs after cross-linking by anti-CD3/NKG2H antibody could be due to reduced cell survival, purified PBMCs were stimulated with immobilized anti-CD3 or anti-CD3/NKG2H antibodies for 48 h and cell survival was measured by Annexin V/7AAD staining (Figure 6A). Remarkably, a significant increase of cell death was observed when activation of PBMCs was carried out in the presence of anti-NKG2H mAb (Figure 6B). By contrast, no significant changes in Annexin V/7AAD staining were observed in PBMCs treated with anti-CD3/NKG2A or anti-CD3/NKG2C antibodies (data not shown). These data clearly show that co-stimulation with anti-NKG2H leads to lower levels of survival than control stimulations with anti-CD3 mAb alone.

### Cell–Cell Interactions, but Not Soluble Factors or MHC Class I Are Necessary for NKG2H Action

The data outlined so far indicate that cultures stimulated with anti-NKG2H antibody showed reduced activation, a decreased proliferative response, and an increased level of cell death. These multiple cellular functions could depend either on soluble factors or they could require direct cell contact.

**Figure 7:**
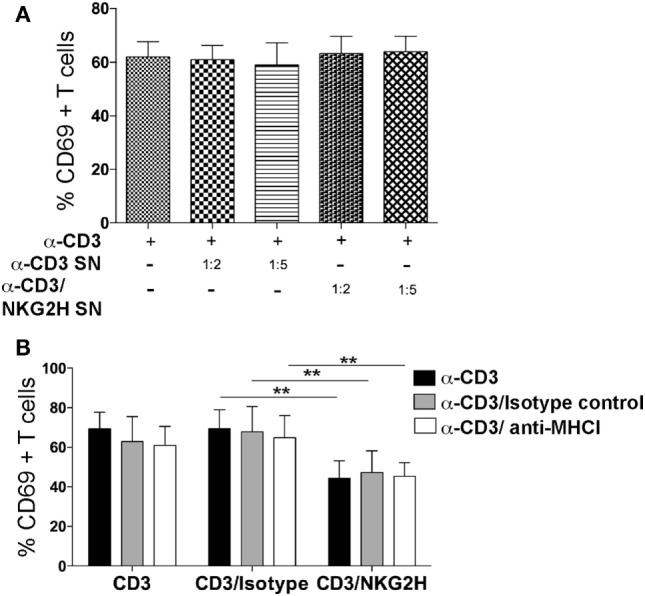
NKG2H suppression of lymphocyte activation is not mediated by a soluble factor and is not blocked by antibodies to MHC class I. **(A)** Supernatants from a culture of peripheral blood mononuclear cells (PBMCs) grown on antibody-coated plates were collected and used at 1:2 and 1:5 dilutions, as indicated, to stimulate a second culture of PBMCs. Cell culture media or the indicated antibodies were used as controls. After 48 h of incubation with the indicated supernatant, cells were harvested, stained for CD69 as a surface activation marker and analyzed by flow cytometry. Leukocytes were gated on forward and side scatter and stained for CD3 and CD69. Mean data and SDs of three experiments with different donors are represented. SN—supernatant collected from cells stimulated with the indicated antibody. **(B)** Freshly purified PBMCs were cultured on plates coated with anti-CD3 (1 µg/ml), anti-CD3/IgG2a control, or anti-CD3/NKG2H (1/10 µg/ml) antibodies for 48 h in the presence or absence of purified MHC class I-specific mAb (clone HP-1F7 at 10 µg/ml). The means and SDs of four experiments with different donors are represented; **p < 0.01.

To investigate the potential role of soluble factors released into the media after anti-NKG2H stimulation of PBMCs, cell-free supernatants collected from cultures after stimulation with either anti-CD3 or anti-CD3/NKG2H antibodies, were added at a range of different dilutions to a new set of cell cultures stimulated with anti-CD3 mAb alone. No differences in the induction of the activation marker CD69 were observed in any of the conditions used (Figure 7A), strongly suggesting that the release of soluble factors was not required for the NKG2H-dependent reduction in cell activation and so, by elimination, cell–cell interactions are probably necessary for NKG2H to elicit the cellular effects shown previously.

Blockade of MHC class I molecules, using the mAb HP-1F7, which detects all classical HLA class Ia, as well as non-classical class Ib molecules including HLA-E (23, 27), did not revert the reduced activation of cells cultured with NKG2H-specific antibody (Figure 7B). These data suggest that MHC class I molecules are not involved in the mechanism of action of NKG2H, consistent with previous data showing that cell surface HLA-E did not trigger cytotoxicity by the K14B06 NKG2H^+^ T cell clone (18).

## Discussion

The NKG2H receptor, a splice variant of NKG2E, was first described as a new member of NKG2 family by Bellon et al. in 1999. However, to our knowledge, no follow-up studies have been published, and presumably it is the lack of specific mAb reagents that has blocked progress in understanding the immunological function of this receptor. The NKG2 family encodes both activating and inhibitory receptors and this function correlates with the presence or absence of ITIM elements in the cytoplasmic tail and the presence or absence of a charged, basic residue in the TM domain that mediates association with ITAM-containing signaling adapter molecules like DAP12. NKG2H is highly homologous, in the cytoplasmic and TM regions, to the NKG2C receptor that associates with DAP12 therefore it was not surprising that DAP12 co-immunoprecipitated with NKG2H. The observation that NKG2H was also able to complex with DAP10 was however, unexpected. Human NKG2D associates with DAP10 and splice variants of murine NKG2D are able to associate with both DAP10 and DAP12 (28, 29). Experiments with chimeric NKG2D receptors and DAP10 and DAP12 signaling adapters have shown that the principal determinant(s) controlling the specificity of adapter association lie in the TM domain (30, 31), but the molecular details controlling receptor–adapter pairing are not known. Inspection of sequence alignments of NKG2H with other members of the NKG2 family has not provided any insights into the promiscuous assembly of NKG2H with both the DAP10 and DAP12 signaling adaptor molecules; thus, further studies will be required to dissect the biochemical basis of this phenomenon.

The availability of a new anti-human NKG2H-specific mAb allowed us to both define the lymphocyte populations that express NKG2H receptor and study the functional consequences of receptor cross-linking. The NKG2H antibody was raised against a specific NKG2H peptide sequence and was shown to specifically stain NKL cells transduced to express NKG2H.

NKG2H was expressed at low levels on the surface of a small fraction of lymphocytes derived from healthy donors. Although some expression of NKG2H was detected on NK cells, this molecule was preferentially present on T cells and, in particular, CD8^+^CD56^+^ T cells. These lymphocytes expressing the NKG2H receptor, were more likely to express CD45RA^+^ than CD45RO^+^; however, there was also a significant population of CD45RA/CD45RO double expressing NKG2H^+^CD56^+^ T cells. CD56^+^ T cells have been reported to express higher levels of NK markers, but not to lyse standard NK-susceptible targets and actually exhibit a relatively quiescent transcriptional profile (32). Thus, overall, our data indicate that NKG2H in healthy individuals is expressed preferentially on naïve/effector cells rather than memory T cells. Previous observations indicate that CD94/NKG2A can be found expressed by both effector and memory T cells, whereas CD94/NKG2C is mainly expressed by highly differentiated CD8^+^ effector T cells (33).

Although NKG2H associates with DAP12 and thus would be expected to act as an activating or co-stimulating receptor, antibody ligation of NKG2H in the presence of suboptimal doses of stimulatory anti-CD3 antibody actually resulted in a profound inhibitory effect on T cell activation in these cultures. By contrast, stimulation of lymphocytes from healthy donors, with either anti-CD3/NKG2A or anti-CD3/NKG2C antibody mixtures had minimal effects on T cell activation in these cultures, indicating that the observed inhibitory effect was specific for NKG2H receptor signaling. These data, together with the flow cytometry details of preferential expression of the NKG2H receptor on a subset of CD3^+^CD8^+^CD56^+^ cells, implies that the T cells activated by NKG2H receptor signaling may negatively regulate the responses of other T cells. In this context, it is tempting to speculate that the CD3^+^CD56^+^CD8^+^CD45RA^+^NKG2H^+^ T cells could be related to a recently identified population of cytolytic CD3^+^CD8^+^CD56^+^CD161^−^ regulatory T cells (CD8^+^ Treg) present in human peripheral blood where the large majority of these cells express the CD45RA marker (34). Further studies are needed to determine how NKG2H-expressing T cells are related to this CD8^+^ Treg subset, which selectively regulates activated CD4^+^ T cells, and the possible implication of the NKG2H expression on these cells, but these experiments will depend on being able to purify and grow NKG2H^+^ T cells *in vitro* for detailed characterization including how the level of receptor cross-linking is related to positive or negative signaling and the downstream signaling events that occur after NKG2H stimulation.

Experiments using culture supernatants collected after anti-CD3/NKG2H stimulation of PBMCs revealed that the suppressive activity could not be accounted by secretion of soluble factor(s) from NKG2H-stimulated cells. A requirement for cell contact for NKG2H to mediate inhibition suggests that these cells act directly on other T cells to prevent activation and interestingly, co-ligation of CD3 and NKG2H was associated with the induction of significant levels of T cell death in these cultures. The simplest interpretation of these data is that the subsets of T cells that express NKG2H negatively affect T cell activation by the induction of apoptosis in bystander responding T cells.

It is still not clear whether this effect is mediated by upregulation of NKG2H expression after TCR-stimulated activation followed by NKG2H ligation and cell intrinsic inhibition or whether the lymphocytes that express NKG2H prior to stimulation become able to inhibit the activation of other T cells *in trans*. Either scenario would explain the failure of multiple attempts to expand populations of purified NKG2H^+^ T cells *in vitro* in the presence of exogenous IL-2 or combinations of IL-2 and the mitogen PHA (data not shown). Alternatively, it cannot be excluded that the proliferative capacity of NKG2H^+^ T cells is limited and/or that specific co-stimuli and/or cytokines are necessary to enable these cells to divide. Such regulation has not been observed for other activating NKR such NKG2C (15, 26), but it would be reminiscent of some features of the CTLA-4/B7 regulatory loop (35).

In this context, it is worth noting that, although DAP12 is generally thought of as an ITAM-containing adapter molecule for activating receptors, its function is more complex (36, 37). DAP12 associated receptors can downregulate TLR-dependent responses in macrophages as well as CD16-dependent responses in NK cells (38, 39). Similarly, DAP12 down-modulates the cytokine production by plasmacytoid dendritic cell *in vivo* during murine cytomegalovirus infection (40) and DAP12-deficient B cells are hyper-responsive after stimulation with anti-IgM or CpG, suggesting that DAP12-coupled receptors negatively regulate B cell-mediated adaptive immune responses (41).

It is worth noting that our observation that NKG2H stimulation triggers inhibition of responses differs from the initial report where aggregation of the putative CD94/NKG2H heterodimer expressed on a T cell clone triggered cytotoxicity and IFN-γ production in a TCR-independent manner (18). This discrepancy might simply reflect that in those experiments receptor cross-linking was done using a CD94-specific mAb and the presence of an activating NKG2C molecule on the clone was never excluded, whereas in our experiments NKG2H was stimulated by a mAb specific for this receptor. It is also possible that adaptations in the T cell clone during the long-term *in vitro* culture necessary for its derivation may have selected for a T cell whose responsiveness may not be representative of the full spectrum of responses of freshly isolated peripheral blood T cells in short-term culture.

No ligands for NKG2H have been identified so far. RMA-S cells transfected with HLA-E and cultured in the presence of peptides that stabilize HLA-E on the surface were not recognized by the T cell clone expressing NKG2H (18). Similarly, in our experiments, addition of an anti-HLA class I mAb (HP-1F7), which detects all classical and non-classical HLA class I molecules including HLA-E (23, 27), failed to completely recover the activation of the PBMCs in the cultures stimulated with anti-CD3/NKG2H antibodies. Finally, although it was suggested that the splice variant NKG2H, might behave similarly to NKG2E and probably bind to HLA-E with similar affinity (16); so far, there are no data that demonstrate an interaction between HLA-E and NKG2H. In aggregate therefore, the available data suggest that it is possible that NKG2H recognizes and binds molecules that are not related, at least to conventional HLA-E-leader peptide loaded complexes. However, our attempts to produce soluble NKG2H molecules, which would greatly facilitate the search for possible ligands for this receptor, have been unsuccessful. Similar difficulties in expression and refolding have also been reported for the ectodomain of NKG2E molecule (16).

In summary, here we have described for the first time the population of cells that express the NKG2H receptor, mainly CD3^+^ T cells, and the effects inserted on lymphocyte function following NKG2H receptor ligation. Although the exact role of NKG2H receptor in T cell activation control remains to be confirmed, the present study suggests that T cells expressing this receptor may act in a negative regulatory circuit to modulate T cell activation.

## Ethics Statement

This study was carried out in accordance with the recommendations of the ethical committee of the CSIC, Madrid, with written informed consent from all subjects. All subjects gave written informed consent in accordance with the Declaration of Helsinki. The protocol was approved by the local ethical committee of the Regional Transfusion Centre, Madrid.

## Author Contributions

DD planned research, performed research, analyzed data, and wrote the paper. DF-S performed research, analyzed data and revised the manuscript. MV-G analyzed data and wrote the paper. HR designed research, analyzed data, and wrote the paper.

## Conflict of Interest Statement

The authors declare that the research was conducted in the absence of any commercial or financial relationships that could be construed as a potential conflict of interest. The reviewer DR and handling Editor declared their shared affiliation and the handling editor states that the process nevertheless met the standards of a fair and objective review.
