# Supplementary material for: NKG2H-Expressing T Cells Negatively Regulate Immune Responses
Source: Front Immunol. 2018 Mar 1;9:390. doi: 10.3389/fimmu.2018.00390 (PMC5837990; doi:10.3389/fimmu.2018.00390)
Supplement: Supplementary file 1 [file image_1.PDF]

Supplementary Figure legends

1. The commercially available anti-NKG2H mAb stains the NKL tumour cell line (that does express NKG2A, NKG2C and NKG2D) only when transfected with NKG2H. A lentiviral vector that co-expresses GFP with NKG2H was used for these experiments and the data are shown as NKG2H expression on GFP positive and GFP-negative cells.

2. Sequence alignment of NKG2A, NKG2C, NKG2E and NKG2H.

3. Representative examples of the staining patterns observed when freshly isolated human PBMC are stained with sera from mice immunised with a peptide sequence specific to NKG2H, but distinct from that used to prepare the commercial mAb. Similar profiles to those obtained in the experiments with the commercial mAb.

4. Preincubation of the commercial anti-NKG2H mAb with an excess of GST-NKG2H fusion protein markedly reduces NKG2H staining of PBMC.

5. Wells of a 96 well plate were coated, in triplicates, with biotinylated anti-CD3, alone or with anti-NKG2H or IgG2a isotype control overnight at 4^oC. After blocking, the amount of anti-CD3 antibody immobilised was visualised using a Streptavidin-HRP secondary reagent.

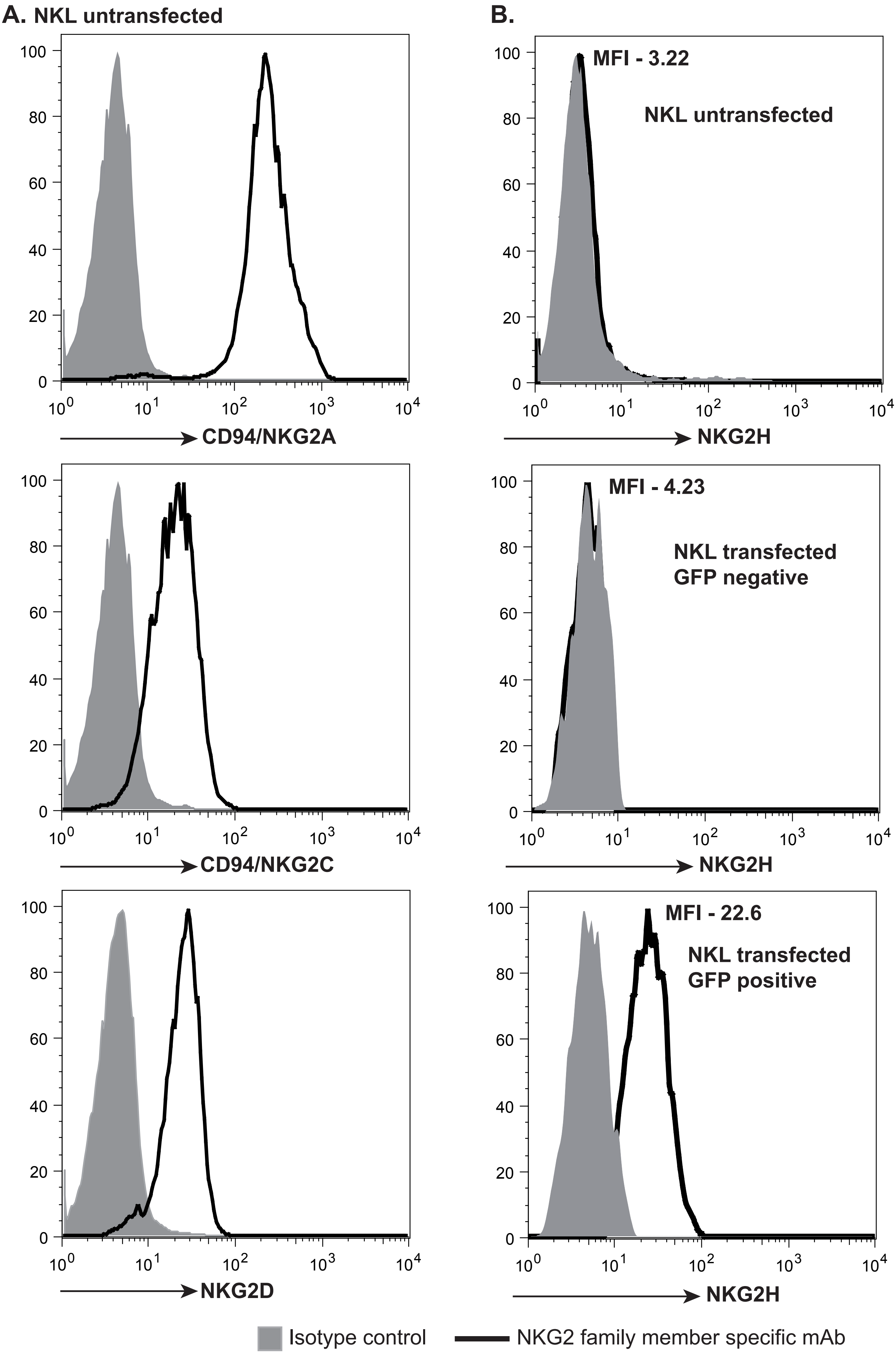
A. NKL untransfected
B.
MFI - 3.22
NKL untransfected
MFI - 4.23
NKL transfected
GFP negative
MFI - 22.6
NKL transfected
GFP positive
CD94/NKG2A
CD94/NKG2C
NKG2D
NKG2H
Isotype control
NKG2 family member specific mAb

Supplementary Figure 2

```
                                      Cytoplasmic Tail

NKG2A      MDNQGVIYSDLNLPPNPKRQQRKPKGNKSSILATEQEITYAELNLQKASQDFQGNDKTYHCKDLPSAPEKLIVG
NKG2C      MNKQRGTFSEVSLAQDPKRQQRKPKGNKSSISGTEQEIFQVELNLQNPSLNHQGIDKIYDCQGLLPPPEKLTAE
NKG2E      MNKQRGTFSEVSLAQDPKPQQRKPKGNKSSISGTEQEIFQVELNLQNASLNHQGIDKIYDCQGLLPPPEKLTAE
NKG2H      MNKQRGTFSEVSLAQDPKPQQRKPKGNKSSISGTEQEIFQVELNLQNASLNHQGIDKIYDCQGLLPPPEKLTAE
           *::*   :*::.*  :** ************ .*****  .*****: * :.** ** *.*: *   **** .

               Transmembrane                   Stalk

NKG2A      ILGIICLILMASVVTIVVIPSTLI     QRHNNSSLNTRTQKARHCG
NKG2C      VLGIICIVLMATVLKTIVLIPFL-     -EQNNFSPNTRTQKARHCG
NKG2E      VLGIICIVLMATVLKTIVLIPFL-     -EQNNSSPNTRTQKARPCG
NKG2H      VLGIICIVLMATVLKTIVLIPFL-     -EQNNSSPNTRTQKARPCG
           :*****::***:*:. :*:    *      .:** * ******** **

           Extracellular CTLD

               β1      β2         α1        β3        α2        β3
NKG2A      HCPEEWITYSNSCYYIGKERRTWEESLLACTSKNS-SLLSIDNEEEMKFLSIISPSSWIGVFR
NKG2C      HCPEEWITYSNSCYYIGKERRTWEESLLACTSKNS-SLLSIDNEEEMKFLASILPSSWIGVFR
NKG2E      HCPEEWITYSNSCYYIGKERRTWEESLQACASKNSSSLLSIDNEEEMKFLASILPSSWIGVFR
NKG2H      HCPEEWITYSNSCYYIGKERRTWEESLQACASKNSSSLLSIDNEEEMKFLASILPSSWIGVFR
           *************************** **:**** **************: * *********

               β4                       β5     β6      β7
NKG2A      NSSHHPWVTMNGLAFKHEIKDSDNAELNCAVLQVNRLKSAQCGSSIIYHCKHKL--------------------------
NKG2C      NSSHHPWVTINGLAFKHKIKDSDNAELNCAVLQVNRLKSAQCGSSMIYHCKHKL--------------------------
NKG2E      NSSHHPWVTINGLAFKHEIKDSDHAERNCAMLHVRGLISDQCGSSRIIRRGFIMLTRLVLNS------------------
NKG2H      NSSHHPWVTINGLAFKHEIKDSDHAERNCAMLHVRGLISDQCGSSRIIVSISFRIKALELAVHQIKFYICSNRNDIMIA
           *********:*******:*****.** ***:*:*. * * ***** *
```

Supplementary Figure 3

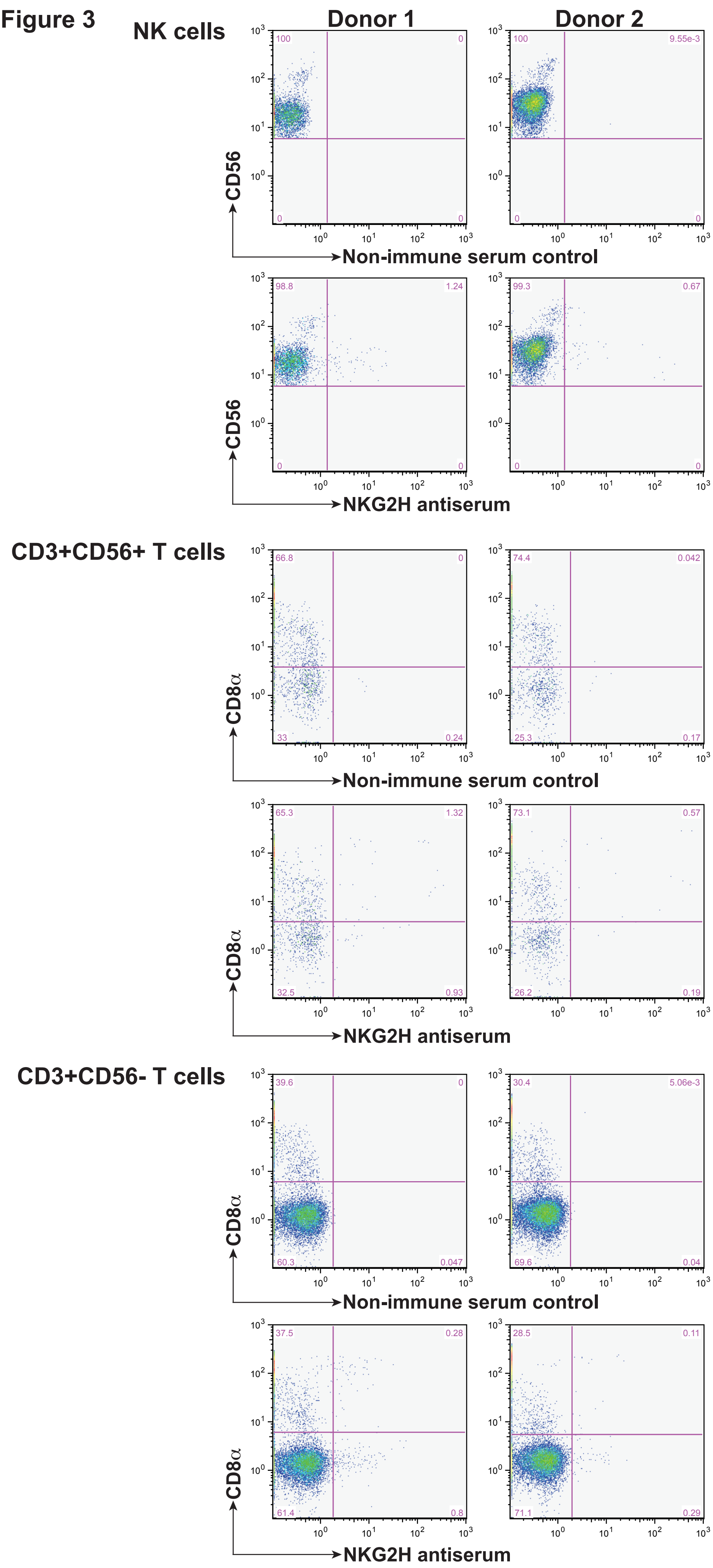

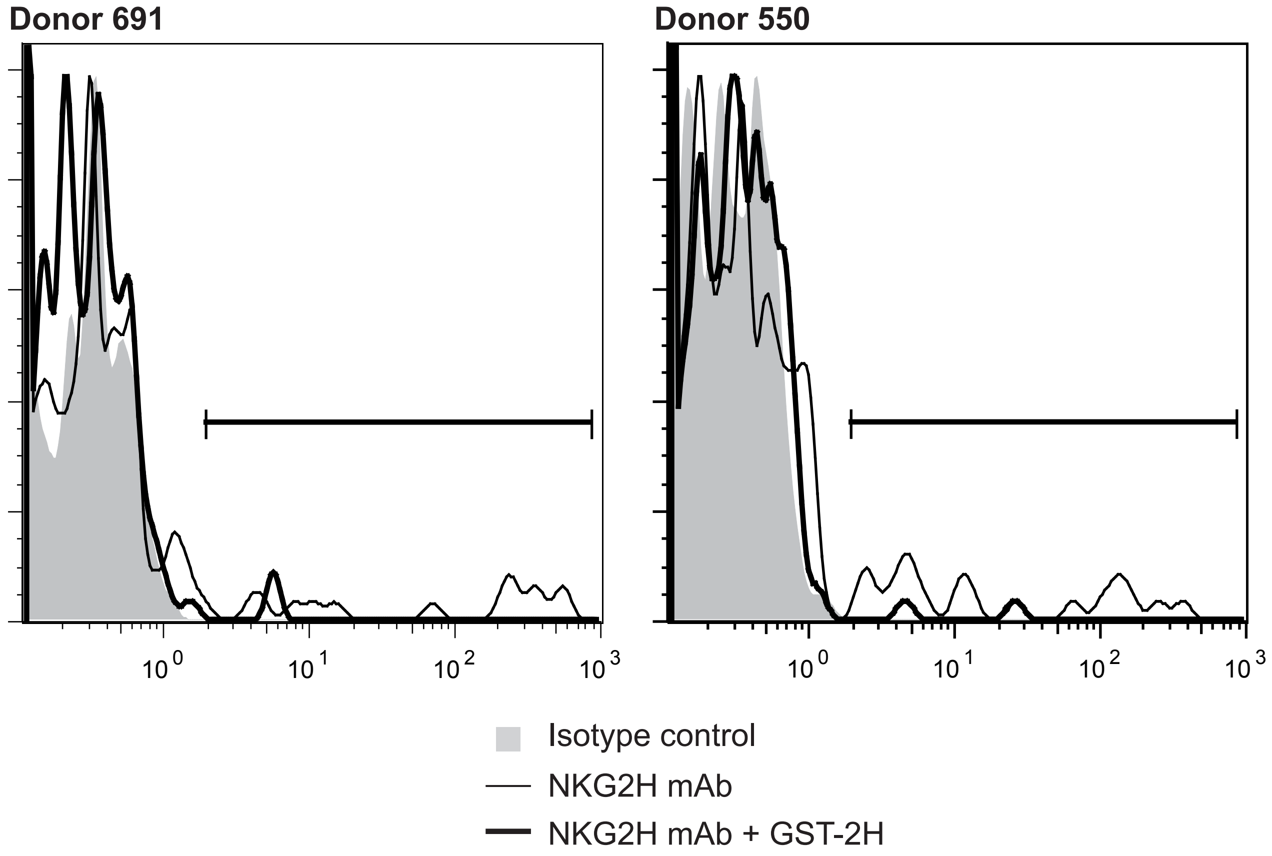
Donor 691
Donor 550
10 0
10 1
10 2
10 3
Isotype control
NKG2H mAb
NKG2H mAb + GST-2H

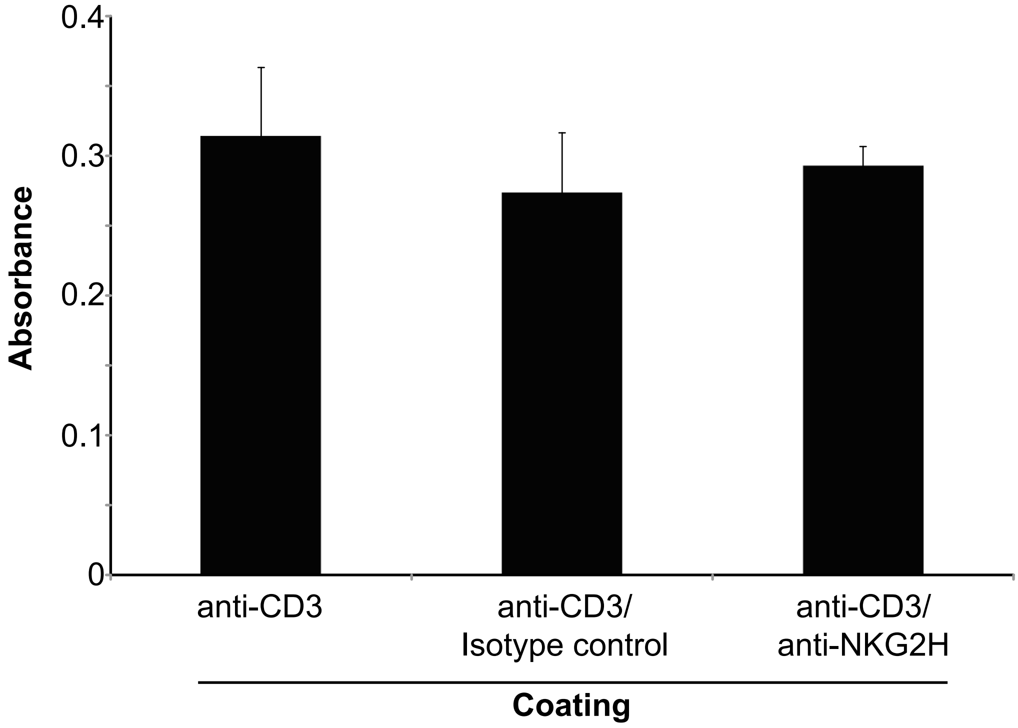

0.4
0.3
0.2
0.1
0
Absorbance
anti-CD3
anti-CD3/
Isotype control
anti-CD3/
anti-NKG2H
Coating
